# Trial of Ovenston; with Remarks

**Published:** 1848-01-01

**Authors:** 


### Medical Jurisprudence in relation to Insanity.

### TRIAL OF JOHN OVENSTON AT THE CRIMINAL COURT, October 27, 1847.

(Before Mr. Justice Maule and Mr. Justice Cresswell.)

John Ovenston, aged fifty-four, upholsterer, was indicted for feloniously shooting George Crawley, with intent to murder him. In other counts the intent of the prisoner was laid to be to maim and disable the prosecutor, or to do him grievous bodily harm.

The prisoner, who still appeared to be suffering from the injury he inflicted upon himself at the time of the transaction, was allowed to be seated during the trial.

Mr. Ryland and Mr. Laurie conducted the prosecution. The prisoner was defended by Mr. Clarkson and Mr. Huddlestone.

Mr. Ryland having briefly addressed the jury, the following evidence was adduced :—

Mr. George Crawley deposed, that he was a wine-merchant, carrying on business in Mark-lane. He first became acquainted with the prisoner in November last, and knew that he was appointed joint assignee with Mr. Cremer in the affairs of a bankrupt named Bond. Witness supplied goods to the bankrupt on their authority, and in January, 1847, a sum of 150*l.* was due to him. It was understood that he was to be paid at the end of two months, and not getting his money he brought an action against the prisoner, and on the result an execution was put into the prisoner's house, and his goods were sold on Saturday, the 14th of August, under a judge's order. On the afternoon of the same day witness received a message which induced him to go to his counting-house, where he saw the prisoner sitting on a chair. He asked him if he wished to see him, and prisoner nodded his head, and witness asked him to step into an adjoining room. He did so, and witness followed him and closed the door. The prisoner then turned round to him and said, "What does this mean?" and before he could reply the prisoner shot him with a pistol. He felt that he was wounded, and rushed out of the room, and was immediately taken to the hospital. He observed nothing particular about the prisoner on this occasion.

Cross-examined: He had only had one previous interview with the prisoner, but he did not then observe anything extraordinary in his appearance. He merely said on that occasion that he could not pay the account, and went away. There had never been the slightest quarrel between them. After witness was wounded he observed the prisoner apparently in the act of taking another pistol from his pocket.

John Yates, clerk to the prosecutor, deposed, that on the afternoon in question the prisoner came to the counting-house about ten minutes to four o'clock. A person named Legg was also present. The prisoner was asked to take a chair while Mr. Crawley was sent for, and on witness leaving the room he observed the prisoner was about to follow him, and he asked him to go back and wait. Witness fetched Mr. Crawley, and he and the prisoner went into a private room together, and in about half a minute he heard the report of a pistol, and cry of murder from Mr. Crawley. Witness ran to open the door, but before he could do so Mr. Crawley staggered out of the room with his face blackened, and he ran into the street. Witness followed him, having previously locked the door, and he immediately heard the report of a second pistol. He afterwards went back to the room where the prisoner was, and found him wounded, and he said, "It was all Mr. Crawley's fault—he had brought it upon himself." He saw nothing particular about the prisoner, but he always considered him a very thoughtful man, and his mind was very intent upon his business.

John Strickland, city police-constable, deposed that from information he received he went to Mr. Crawley's, and he was informed by Yates that his master had been shot by a man who was in the counting-house. He found the prisoner leaning his head in his hands, and bleeding from the mouth. A pistol was lying by him, the muzzle of which was covered with blood, and quite warm, as though it had been recently discharged. He fired a second pistol in the room, and when he was picking it up, the prisoner said it was that —— villain Crawley had caused it all, and he said he was very sorry the ball had not gone through his (prisoner's) head. He then asked if Mr. Crawley was hurt, and also said that he had always endeavoured to get his living honestly, and he had been wrongly done by.

Cross examined: It was in the room that the prisoner said it was that —— villain Crawley had done all this, and that he also inquired how Mr. Crawley was going on. He made the same observation when they were in the cab going to the hospital. The prisoner inquired very anxiously and kindly about Mr. Crawley, and he appeared at that time quite quiet and collected.

Mr. Thomas Cremer deposed that the paper produced was in the handwriting of the prisoner.

The paper was put in and read. It was taken from the prisoner by the constable at the time he apprehended him. It was addressed to his sisters, Mrs. Mary Dickson and Beatrice Jenkinson Ovenston, and went on as follows:—"Mr. Dawson has the painting of Jairus' Daughter as security of the money I had on loan from him, which I wish to be settled, and intended to do so as soon as I got settled with D. Walker; but Mr. Sheard says he will try and claim the statute of limitations. This will show that he is a villain at heart if he does, and ought to be exposed to the world at large. There are debts owing to me much greater than I owe, which can be collected, and every one paid that I justly owe, and there will be a surplus which will belong to you both. The disgrace which has been brought on me by that villain Crawley is from my not consenting, with him and Cremer, to get possession of the Bull Inn, which I could not do; and I saw that there was something underhand going on between him and Bond. When Cremer requested me to allow some articles to be sent in to the Bull Inn, he said they might come to 30*l.*, and he and Crawley made a very different affair of it, but it would end in no good to either of them—one must go for an example to deter such wretches from doing the like again. Death is far more preferable than the disgraceful situation that villain Crawley has placed me in, and he has driven me to do that which I never contemplated. This action will, no doubt, cause great grief to you all, but you must forgive it, as you could never expect that I could ever know happiness again in this world after this villain Crawley's conduct towards me, and to see me pine away day after day I knew would break your hearts. Therefore, console yourselves in the best way you can, and I trust you will meet with kind-hearted friends who will assist and console you in your affliction, and that time will wear smooth that which now appears so rough and rugged to your views and feelings. Remember me to poor old Mr. Jacobs. I hope that Mrs. Drummond will keep her promise, and get your nephew Johnny into Christ's Hospital. I know that if she can get a presentation she will do it.

(Signed) "J. O."

Mr. William George Legg deposed that he was in the counting-house of the prosecutor when the prisoner came there, and he gave a statement of what occurred in the same terms as the witness Yates. He observed nothing particular in his appearance. He asked him what o'clock it was, and he replied, "No, no, I have left my watch at home." He always thought the prisoner an excitable man.

Mr. James Riley, clerk to another wine merchant in Mark-lane, proved that upon hearing the alarm he went to Mr. Crawley's, and saw the prisoner, who was wounded. Witness asked him what could have made him do such a thing, and he replied, "Crawley has ruined me! that is the reason I shot him." Immediately afterwards he said, "How is poor Crawley? I am very sorry I shot him; I hope you have got good advice for him." Witness told prisoner he had shot himself outside his head, and he replied that he intended the ball to have gone through.

Mr. F. Nicholls, merchant, in Mark-lane, said that he also went to

Mr. Crawley's counting-house, upon hearing the cries of murder and police, and found the prisoner in the condition above stated, and he heard him say, when the policeman picked up a pistol, that the —— thing had not done its work, referring to himself, the ball having lodged in his head.

Edmonds, another City constable, who assisted in taking the prisoner to the hospital, deposed that while he was searching him, to see if he had any more fire-arms, he threw his purse upon the ground, and said, "By —— I have not!" As they were going to the hospital, the prisoner put his hands down, and said, "I am quite prepared." Witness afterwards saw a pistol ball extracted from the prisoner's head.

Mr. Francis Kearsey, attorney for Mr. Crawley, produced the proceedings in the action brought by him against the prisoner. The cause did not go to trial, but the defendant assented to a judge's order, undertaking to pay debt and costs on a certain day, and in default confirming judgment and execution. Default was made and execution issued on the 14th.

Mr. Harris, a surgeon, in Fenchurch-street, deposed that he examined the prosecutor after the occurrence, and found he had a bullet wound in the jaw. He wished to go to the hospital, and witness told him he might do so with safety. He afterwards saw the prisoner, and felt a pistol ball inside the skin of his head, which appeared to have entered under the left cheek. The prisoner said to him, "How is poor Crawley?" Witness told him he thought he would do well, and the prisoner made no answer. He afterwards asked the prisoner if he had shot himself, and he said he had, and he was very sorry the ball had not gone into his brain. He appeared very dejected. The wound received by Mr. Crawley was a dangerous one.

Cross-examined: In witness's opinion, the prisoner put the pistol into his mouth when he discharged it.

Mr. Solly, senior assistant-surgeon at St. Thomas's Hospital, deposed that he saw Mr. Crawley on the evening of the 14th August. On examining him he found a gun-shot wound in the cheek, near the nose, and he afterwards extracted a bullet from the wound. It had gone straight along the upper jaw, raking along the fangs of the teeth, and entered a depth of two inches and a half, and was embedded in the bone a quarter of an inch. The wound was most certainly one dangerous to life.

By the Court: Mr. Crawley remained in the hospital from three weeks to a month.

Mr. M'Murdo, surgeon to the gaol of Newgate, and also of St. Thomas's Hospital, deposed that the prisoner was under his care, both at the hospital, and in the gaol after his committal. He had seen him continually, and noticed particularly his conduct and demeanour, but never observed anything to lead him to believe that he was of unsound mind.

Cross-examined: The prisoner was very much depressed, but he should consider that he was in general a reserved man. Any great losses or distress would have effect upon the mind of a man partially affected by insanity. If a person committed an act of violence from insanity produced by such a cause, he should expect that it would last some time, and that the party would not appear quite calm and collected immediately afterwards.

Mr. Charles Holding, deputy-surgeon, gave similar testimony to that of Mr. M'Murdo, and said, that from all he had seen of him there was nothing to induce him to believe that the prisoner was any other than of sound mind.

This was the case for the prosecution.

Mr. Clarkson then addressed the jury for the defence. He said that a most painful duty was cast upon himself and his learned friend, in having to defend the unfortunate man at the bar; and the jury would have observed, by the course taken in cross-examination, that they never for a moment attempted to deny that it was his hand that committed the act; but they did hope to satisfy the jury that at the time it was committed the prisoner was in such a state of mind as not to render him criminally responsible for the act, of which he was undoubtedly guilty. If he should succeed in establishing that fact to their satisfaction, he reminded them that it would not have the effect of relieving him from all punishment, but he would be placed in such safe custody as would render it impossible for him to do any violence either upon himself or any other person in future. The learned counsel then proceeded to comment upon the circumstances of the case, and he put it to the jury whether, if the desperate attempt made by the prisoner upon his own life had been successful, and instead of being in their present situation, they had been called upon to assist the coroner in an inquiry as to the means by which he met his death, they would have had any difficulty in coming to the conclusion that at the time he destroyed himself he was in a state of temporary derangement? The sole question they were called upon to decide was, the state of mind of the prisoner at the time he committed the act; and if he should show them by undoubted testimony that during the whole of his life the prisoner had been remarkable for his kind and humane character, and that his mind had been overwhelmed by the distresses and misfortunes that had come upon him, the climax of which was the sale of all he possessed, under the execution, and which had completed his ruin, he did not think the jury would find any difficulty in coming to a conclusion that it was during a temporary paroxysm of madness, thereby occasioned, that he committed the act with which he was now charged. He was ready to admit that almost immediately afterwards the reason of the prisoner returned to him, and that at the present moment he was perfectly sane; but this did not in any way affect the proposition that he had submitted to the jury, that at the time when the act was accomplished he was not in such a state of mind as to be responsible, criminally, for what he did. The learned counsel then read extracts from several works upon the subject of insanity, to show that in a great proportion of cases it was occasioned by pecuniary difficulties or domestic affliction, and that the number of cases of insanity arising from moral causes was infinitely greater than from physical ones; and he said he should satisfy the jury, by the evidence of Dr. Conolly, the principal physician of Hanwell Lunatic Asylum, and other eminent men, that this was the case, from their own experience; and they would also state to them that, in their opinion, the mind of the prisoner was temporarily destroyed by the prisoner's difficulties, and the sale of his property under the execution; and that they had no doubt that at the time the prisoner made the attack upon Mr. Crawley, he was not at all aware of the consequences of what he was about to do. Mr. Clarkson then proceeded to detail the nature of the evidence he should adduce, and the following witnesses were examined :—

Sir John Easthope deposed, that he had known the prisoner more than twenty years, and had continually employed him as an upholsterer. He always thought him a sensitive man, but particularly kind and humane.

Mr. William Tite, the architect, who had known the prisoner five-and-twenty-years, said he had the highest opinion of the prisoner's kindness and benevolence. Twenty years ago he and witness were appointed assignees to an estate which was indebted to the prisoner 900*l.*; and, notwithstanding his loss, he purchased all the plate and linen at the sale, and handed them over to the lady of the gentleman who was indebted to him; and a similar case occurred within the last four years. His conduct was most benevolent in every respect—he endeavoured to get children into schools, and exerted himself in other benevolent proceedings. About two months before this transaction, the prisoner came to him in a very excited state, and consulted him with regard to the sale of his library.

Mr. W. J. Hall described the prisoner as the most kind, amiable, and humane man he had ever met with. He had also been co-assignee with him in a case where a debt had been contracted with him under aggravated circumstances, and where he behaved with a kindness seldom met with in commercial transactions. For some time he had observed a great change in his conduct; he appeared wild and incoherent, and, in fact, he considered his mind was decidedly affected, and that he was unfit to manage his concerns. The prisoner had frequently spoken to him of his connexion with Bond and Cremer, but he never could make head or tail of what he said upon the subject.

Mr. T. Tilleard, the late under-sheriff, spoke highly of the prisoner's character. Witness's father was solicitor in the bankruptcy referred to by the last witness, and knew that the prisoner lost a thousand pounds by that transaction.

Mr. Shear, clerk to an attorney, had known the prisoner several years, and considered him a man of unusually kind disposition. Witness had acted professionally for the prisoner, and knew that he had sustained heavy losses, several of them almost simultaneously, and he observed a great change in his conduct afterwards, and did not appear to understand the advice that was given to him, or if he did, he could not act upon it, and he also observed a great change with regard to his memory. He also said frequently, with great energy, there must be a conspiracy among some people to ruin him. Mr. Bond was an intimate friend of the prisoner, and he lost 600*l.* by the bankruptcy, a large sum by the sale of his furniture, and became responsible to the extent of 500*l.* as the assignee. During the nine months prior to the transaction, the prisoner had lost in debts from intimate friends more than 2,000*l.*, and this was independent of the transaction with Bond.

Mr. H. B. Hall had known the prisoner twenty-two years. Latterly he had observed a decided difference in his conduct, and he exhibited strong excitement, apparently brought on by his troubles, and his mind was so affected that he could not cast up three lines of figures correctly. In addition to his pecuniary troubles, a lady friend of his committed suicide last November, and he frequently alluded to the circumstance, and it seemed to prey upon his mind. Since the year 1844 the prisoner had sustained losses to the amount of 3,800*l.* A short time back the prisoner used frequently to write upon large placards the words, "Statute of Limitations Act here;" and this referred to the circumstance of an intimate friend who owed him a large sum of money having pleaded that statute to an action brought against him. He frequently appeared wild and raving, and on the 12th of August his conduct was not that of a reasonable being, and he kept running in and out of the house. Witness saw him on the morning of this unfortunate transaction, and he appeared haggard and worn out, and said that he had passed a sleepless night, and burst into tears.

Mr. J. H. Utting deposed that he had known the prisoner for twenty years. He had latterly observed a very material alteration in him. Saw him in June, 1847, upon business, and found him quite a different man to what he used to be, and his memory continually failed him, and he did not appear to know what he was about. Witness had been employed by the prisoner, and he would frequently order him to do things and immediately afterwards he would countermand the order, and acted in a most extraordinary manner.

Another person, named Hope, who had also been employed by the prisoner, gave similar testimony to that of the last witness. According to his opinion, on several occasions the prisoner exhibited a total failure of memory.

Mr. Henderson said he had known the prisoner between forty and fifty years, and he always considered him a most benevolent, kind man, particularly to those in distress. He met the prisoner by accident about three o'clock on the 14th of August, and he observed a very great alteration in his appearance, and particularly in the expression of his eyes.

Mr. Blackburn had known the prisoner twenty years, and he also said that he had latterly observed a very great change in his conduct, which was very different to what he ever saw before.

Mr. T. Dixon, the prisoner's brother-in-law, said he had known him for twenty-two years, and that after his misfortunes came upon him there was a great change in him. His memory failed him. His nights were restless, and he appeared quite worn out. On the day his property was sold under the execution, witness asked him whether he wished him to purchase any of the articles, but he evaded the question, and gave him no answer.

Mrs. Downey, the prisoner's sister, gave evidence of a similar character. On the day of the attack made by the prisoner he did not eat any breakfast, and appeared very melancholy and desponding, and said a great many things which she could not understand.

Miss Mary Ovenston, another sister, deposed to the same facts. She said that prior to August last her brother was kind, and cheerful, and humane, and after that period he became morose and excitable, and she had consulted a friend as to the propriety of having him placed under restraint.

One or two other witnesses were examined, who deposed to circumstances of a similar character.

Dr. Conolly was then called as a witness. He deposed, that he was chief physician to Hanwell Lunatic Asylum, and had besides the care of a private establishment of his own, and had devoted his entire attention to diseases of the mind, and at the present time he had upwards of a thousand patients under his care. He had heard the whole of the evidence in this case, and had also had an interview with the prisoner in Newgate, and he had applied his attention and experience to all the circumstances, and the impression conveyed to his mind from all the facts was, that at the time the prisoner made the attack upon Mr. Crawley he was not in a sound state of mind. He had known many cases where pecuniary losses and domestic affliction had deprived the mind of reason. This happened, in fact, in innumerable cases, and they were more frequently the causes of insanity than any others of a physical nature. In his opinion the mind of the prisoner had been gradually losing its power from the difficulties in which he felt himself surrounded, and that the crisis had arrived when he committed this act; and he did not consider that his being at the present time, or very soon after the transaction, in a state of perfect sanity, in any way affected the opinion he had formed, or was at all inconsistent with that view of the question. Dr. Conolly added, that at the present time he was acquainted with several perfectly analogous cases.

Cross-examined: He did not go the length of saying that he was unconscious of what he did, but he believed he was acting under an impulse which he could not control, and that he could not distinguish the wickedness of the act, although he was conscious that he was committing it.

Baron Maule: Then you are of opinion that at the time he was not capable of knowing that he was committing a guilty act.

Dr. Conolly: I think so.

Dr. Forbes Winslow and Mr. Streeter, surgeon, were also examined, and they concurred entirely in the view of the case taken by Dr. Conolly.

Baron Maule then briefly summed up, and the jury, after deliberating a short time in the box, expressed a wish to retire. They were absent for half an hour, when they returned into court and gave a verdict of not guilty, on the ground of insanity.

The prisoner was ordered to be detained during her Majesty's pleasure.

---

### REMARKS ON OVENSTON'S CASE.

BY THE EDITOR.

This case has been made the subject of much comment in the public journals of the day, and the medical men who were called upon to give evidence in favour of the plea of insanity, have been held up to censure for the view they took of Ovenston's state of mind at the moment he committed the offence which brought him before the judicial tribunal of his country. Dr. Conolly has particularly been referred to, and his examination has been canvassed with great freedom. It is not our intention to question the right of journals not strictly professional in their character, to discuss, with almost unrestricted liberty, the opinions which any scientific man may give in elucidation of matters which are made the subject of investigation in our criminal or other courts. These criticisms ought, however, to be penned with much caution, particularly when they have reference to points so extremely difficult of satisfactory solution as those connected with the morbid phenomena of the human mind, in relation to the degree of responsibility and knowledge of right and wrong existing at a given moment. Again, it is not exactly fair to the medical witnesses to judge of the amount or description of evidence submitted to them for consideration, and upon which they have based their opinion, from the necessarily abridged accounts reported in the papers of the day. The evidence in Ovenston's case, in all its minute details, was submitted to the medical witnesses, and many very interesting and important particulars came under their notice, which did not appear in the report of the trial, and which, in fact, were not brought at all under the cognizance of the court. The particular facts referred to certainly threw a great light on the case.

It should be recollected, that in the cases in which the plea of insanity is urged in extenuation of crime, the medical witnesses often stand in and peculiar a painful position. Upon the value of such evidence there cannot be, among liberally minded and intelligent persons, two opinions.

Questions of great difficulty and complexity may arise in the course of an important trial connected with science, upon which the judge, jury, and counsel may be incompetent to form a sound opinion. With a view to the elucidation of the matter, men who have made the question at issue a special object of study are called upon to solve the disputed point, and their evidence is generally considered as conclusive. This we conceive to be the proper view to take of the matter. In a case in which it becomes necessary, for the purpose of satisfying the ends of justice, to submit certain portions of food, or the contents of the stomach, to careful chemical analysis, in order to ascertain, by the aid of delicate tests, whether a party had come to his death by fair or foul means, persons who have a reputation for having paid particular attention to such investigations, and who are practical chemists and toxicologists, are, as a matter of course, called upon for their opinion, and upon the result of their investigations the life or death of a fellow-creature often depends. No man in his senses disputes the credibility and great importance of such testimony. A similar course is pursued when any difficult and complicated question arises connected with mechanics or civil engineering. The most scientific men of the day are summoned to solve knotty points, and to settle questions of disputed science, which experienced men only can satisfactorily set at rest. For what purpose are questions of great legal difficulty and subtlety submitted to the adjudication of the judges assembled in the highest courts in the kingdom? Is this not deferring to the judgment of men of known experience, sagacity, and extensive legal knowledge? This being the case, we cannot conceive why medical men, whose lives have been devoted to the study of the diseases of the mind, should not be equally competent with the experienced mechanician, the celebrated practical engineer, the learned jurist, the scientific chemist, to pronounce *ex cathedra* on points coming strictly within their own peculiar province, and to the study of which they have devoted the best portion of their days.

The cant of the day is, whenever a medical man is placed in the witness-box to give evidence in these cases, "Oh, he is a mad doctor!" "He is a keeper of an asylum!" "He has an interest in making people out mad!" Such puerile declamations are unworthy of intelligent and thinking men; it may suit the purpose of a counsel who is not over anxious to investigate the truth, and not over scrupulous in the means he makes use of to effect his object and promote the interest of his client, to say to the jury, "Why, gentlemen, these 'mad doctors' want to make us all out insane. Take care, gentlemen; one of you may be the next victim; and some trifling eccentricity—some little waywardness—some evanescent weakness—some foolish or indiscreet admission—may consign one of you to those dreary regions of despair, upon the portal of whose dominions is inscribed the fearful announcement that he—

"'Who enters here leaves hope behind.'"

Medical witnesses are, alas! but *men*, and are therefore liable to the infirmities of human nature. We must, however, do the profession the credit of saying, that notwithstanding the unfair examination to which an unscrupulous advocate may sometimes subject them in our courts of justice, they generally come out of the ordeal with a very good grace.

Again, it should be recollected that when a medical man is summoned to give his opinion in a court of law upon a case in which it is important to ascertain the degree of sanity that existed at any stated period, he gives his opinion upon an abstract point, to the best of his knowledge and ability, *without any reference to ulterior results*. He has nothing to do with the consequences of his evidence, whether life be prolonged to an indefinite period, or whether a fellow-being shall be immediately launched into eternity. To the question, "Do you consider A, B, or C insane? was he so, according to the sworn testimony of credible witnesses, at such a period?" the medical gentleman, experienced in the characteristics of insanity, answers "yes," or "no," according to the best of his knowledge and judgment. If the accused party escape punishment,—if, in consequence of the medical evidence, his life be saved, we do not see a semblance of justice in holding the medical witness up to public odium and censure. They are not the parties who have made the laws, (be they bad or good,) neither are they to be held accountable for the escape of the prisoner from the extreme penalty of the law. They are sworn to state the truth according to their honest convictions, *malgré* the consequences. If the law be defective, let the legislature alter the penal code; if insane people are to be hanged like dogs, let those who maintain such a doctrine take steps to force their views upon the legislature of the country.

But to the case of Ovenston. We opine never was a clearer case of derangement of mind made the subject of judicial inquiry. It has been said, that supposing his mind to have been thrown off its healthy balance by the great misfortunes and anxiety with which he had to combat, he was a fit subject for punishment, because he attempted the life of a man against whom there existed a *real cause for enmity.* This fact does not alter our view of the case. Madmen redress real grievances as well as imaginary ones. The fact of a person shooting an individual who had by any course of conduct excited his animosity, does not prove either sanity or responsibility. Parties experienced in the management of the insane, and who are practically acquainted with their characteristics, know that it is a common occurrence for persons palpably, unequivocally, and often outrageously insane, to resent any injuries which they may have received from others. The late Dr. Haslam relates an instructive case of this kind:—A patient who was confined in the Manchester Lunatic Asylum had been subjected to very cruel treatment, and in consequence of it, he killed the person who had the care of him. He related, with great calmness and self-possession, the particulars of the transaction to Dr. Haslam. He said, "The man whom I stabbed richly deserved it. He behaved to me with great violence and cruelty; he degraded my nature as a human being; he tied me down, hand-cuffed me, and confined my hands much higher than my head, with a leathern thong; he stretched me on the bed of torture; after some days he released me. I gave him warning; for I told his wife I would have justice of him. On her communicating this to him, he came to me in a furious passion, threw me down, dragged me through the court-yard, thumped me on my breast, and confined me in a dark and damp cell. Not liking this situation, I was induced to play the hypocrite. I pretended extreme sorrow for having threatened him, and by an affectation of repentance, prevailed on him to release me. For several days I paid him great attention, and lent him every assistance. He seemed much pleased with the flattery, and became very friendly in his behaviour towards me. Going one day into the kitchen, where his wife was busied, I saw a knife: this was too great a temptation to be resisted: I concealed it about my person, and carried it with me. For some time afterwards, the same friendly intercourse was maintained between us; but as he was one day unlocking his garden door, I seized the opportunity, and plunged the knife up to the hilt in his back." "He always mentioned this circumstance," says Dr. Haslam, "with peculiar triumph, and his countenance (the most cunning and malignant I ever beheld) became highly animated at the conclusion of the story." *Ex uno disce omnes.* To the question, whether insane people do not exhibit revengeful feelings towards parties who have inflicted real injuries upon them, there can be but one answer among practical and experienced men.

Ovenston was a man naturally of a cheerful, confiding, amiable, and benevolent character. He had, by an overweening confidence in others, suffered heavy pecuniary losses. One difficulty followed rapidly on the heels of the other, embarrassment succeeded embarrassment, until his mind became completely bewildered and almost quite prostrated, as a consequence of these heavy pecuniary losses. Those more immediately about the man noticed a marked change in his natural character. From being a careful and discreet man, he became reckless and unreasonable,—from having the most implicit confidence in his friends and legal advisers, he manifested great self-will, perverseness, and obstinacy. He gave orders to his solicitor, and almost immediately countermanded them, without appearing to have any clear views as to his proceedings. With this he was subject to great depression of spirits, and bodily ill-health, complaining constantly of an inability to sleep. This sleeplessness, which all the witnesses swore to, and which was a peculiar feature in his case, is considered to be a peculiar and characteristic indication of the early stage of disturbed or insane mind. He was said to have exhibited "worn-out looks," and to manifest an evident and most extraordinary capriciousness, accompanied with an inability to exercise a continuity of thought or action on any one point. The witness Downey swore to a "marked alteration of manner" in Ovenston. This witness, in the course of her evidence, made use of a remarkable expression as characteristic of Ovenston's state, a few days before he shot Mr. Crawley, which conveys, we think, a most graphic description of an insane man. She swears that on the day when the attempt at assassination took place, Ovenston was "*unusually excited, and never at rest!*" How truly descriptive of a disturbed and unsound mind somewhat in advance of the stage of *incubation!*

Ovenston's bodily health was evidently failing, *pari passu,* with his derangement of mind. He lost his appetite, and the merest trifles annoyed and excited him. He was easily irritated, and liable to sudden bursts of angry emotion. He manifested a feeling of suspicion, quite opposed, as the witness said, to his former almost "blind confidence" in those who addressed him. His spirits were noticed to be much depressed, and this depression alternated with great excitement of manner and conversation. At one time, whilst walking in St. Paul's Churchyard with a friend, a conversation took place in relation to his affairs, and Ovenston exhibited such violence of conduct, as to attract the notice of the passers-by. He would, when in these paroxysms, stamp his feet, and tear his hair. With the want of sleep, of which he was ever complaining, he exhibited "a feverish state of the body." His memory failed — he forgot the names of his most familiar friends — misdirected letters, and Mr. Hall swore to his manifesting a "melancholy state of mind, alternating with violent bursts of passion." His knowledge of accounts failed him. He was originally an excellent and accurate accountant. In this particular his mind had undergone a remarkable alteration—the most simple arithmetical questions set him at defiance. With all these indications of an insane state of mind, Ovenston believed in the existence of a conspiracy. His mind dwelt much upon this point. Contrary to all his usual mental characteristics, he manifested a most unusual and extraordinary confidence in persons who were, comparatively speaking, strangers to him. He was naturally a cautious and prudent man, particularly in all monetary matters, carefully weighing the amount of reliance to be placed in each individual with whom he was brought into association. In this particular his character underwent a remarkable alteration. He lent large sums of money in a most reckless manner to strangers, without a prospect of repayment. Again, according to Miss Ovenston's evidence, he became very angry at trifles, and on the day when he committed the offence for which he was tried, he looked sad and wept. The preceding is an abstract of the evidence submitted to the medical witnesses as data upon which to form on opinion of Mr. Ovenston's state of mind prior to the commission of the alleged crime. We had, in conjunction with Dr. Conolly and Mr. Streeter, an eminent and clever general practitioner, an opportunity of conversing with Ovenston in Newgate, a few days before his trial at the Central Criminal Court; he then appeared in a composed and sane state of mind. His own account of the transaction is as follows. He says, when he called upon Mr. Crawley, he did so without having any definite object in view. He was not conscious of anything about him, his mind being in a dreamy state. After firing at Mr. Crawley, and then shooting himself, he lost a large quantity of blood. Immediately following this hæmorrhage, Ovenston's mind appeared to have regained its consciousness, and he became sensible of the dreadful situation in which he had placed himself. To use his own emphatic expression, his brain was relieved of a great pressure. We can well understand the rationale of this. The anxiety and distress of mind to which he had been so long subjected, had produced an amount of cerebral congestion and fulness of the vessels of the brain, which had evidently been interfering with the healthy manifestations of the mind. It is a matter of common occurrence for a person labouring under a temporary paroxysm of insanity, accompanied with a tendency to suicide, to be restored to the healthy use of his reasoning powers as the result of the loss of blood following an unsuccessful attempt at suicide. Such appears to have been the effect upon Ovenston's brain. The above are the grounds upon which we, in conjunction with the other medical witnesses, came to the conclusion that this man, the moment he committed the act, was not a responsible agent, and was unable to distinguish between right and wrong.

---

